# Seroprevalence and Knowledge of Hepatitis B Virus Infection Among LaboratoryWorkers at Kilimanjaro Christian Medical Centre in Moshi, Tanzania

**DOI:** 10.24248/EAHRJ-D-16-00362

**Published:** 2017-07-01

**Authors:** Richard E Machange, Dominic Mosha, Jeremia J Pyuza, Balthazar B Nyombi, Elichilia R Shao

**Affiliations:** a Department of Internal Medicine, College of Medicine, Kilimanjaro Christian Medical University College, Moshi, Tanzania; b Department of Clinical Laboratory, College of Medicine, Kilimanjaro Christian Medical University College, Moshi, Tanzania; c Department of Community Medicine, College of Medicine, Kilimanjaro Christian Medical University College, Moshi, Tanzania; d Better Human Health Foundation, Moshi, Tanzania; e Imagedoctors International, Arusha, Tanzania

## Abstract

**Background:**

Hepatitis B Virus (HBV) is transmitted through blood, infected body fluids, and unsterile needles and surgical equipment. We first determined the current seroprevalence of HBV and vaccination coverage, then assessed knowledge on risk factors for hepatitis B virus infection among medical laboratory workers at Kilimanjaro Christian Medical Centre (KCMC) in Moshi, Tanzania.

**Methods:**

A cross-sectional study was conducted from January to June 2014, involving health care workers (HCWs) from KCMC. Eligibility for participation in the study was determined by length on employment, provision of consent, and willingness to complete a questionnaire. Recruitment was non-randomised; a simple and consecutive sampling of 85 eligible HCWs was conducted at the hospital during study period. Structured questionnaires were self-administered and consenting participants allowed blood samples to be tested for HBV infection. Blood (4 mL) was obtained by vene-puncture from all participants using sterilised disposable 5 mL syringes and 20 gauge needles. All collected blood was tested for HBsAg using enzyme linked immunosorbent assay according to manufacturer's guidelines. A cut-off point of 10% (*P*>0.1) was used to select variables to be included in the further analysis.

**Results:**

Out of the 76 HCWs eligible to participate in the study, only 8 (10.5%) were vaccinated against HBV. Of the 68 unvaccinated laboratory workers, 9 (11.8%) tested positive for HBsAg. Knowledge about HBV infection and its associated risks was high among medical personnel – where 36.4% scored above 65% – compared with non-medical personnel, none of whom scored as high as 65%.

**Conclusion:**

Seroprevalence of HBsAg among laboratory workers at KCMC was 11.8%. Low knowledge of risks for HBV infection placed HCWs at great risk of occupational exposure. Low vaccination coverage among HCWs increases the risk of acquiring HBV infection following occupational exposure.

## BACKGROUND

Hepatitis B virus (HBV) infection is among the viral diseases responsible for serious liver diseases, such as cirrhosis and hepatocellular cancer. Viral hepatitis is a serious public health problem affecting billions of people. Globally, approximately 240 million people are chronically infected with HBV.^1,2^ The prevalence of HBV has been reported to be higher in East Asia and sub-Saharan Africa.^1,2^ In countries with high HBV prevalence, most HBV infections happen during childhood. Of the children who are not infected, many remain at risk for contracting the disease as adults, if they are not vaccinated.^3^ For health care workers (HCWs), the risk of contracting HBV is much higher than the general population, due to the nature of the work they do daily.^4^ A study in Niger demonstrated that HCWs have 4 times the risk – compared with the general population – of becoming infected with HBV through direct contact, including needle sticks with infectious materials like HBV-infected blood, blood products, or contaminated fluids.^5^

In developing countries, 50% of HBV infection among HCWs has been attributed to professional hazard.^6^ Studies conducted in Pakistan and Nigeria indicate that poor knowledge, leading to inadequate precautions against blood-borne infection; lack of adequate training on infection prevention control of hospital-acquired infections; and low coverage of vaccination against HBV, among others, contribute to the high prevalence of HBV among HCWs.^7,8^

Through vaccination, HBV infection is a preventable disease. Despite the availability of HBV vaccines, HCW vaccination rates are not sufficient and significant numbers of HCWs are unvaccinated or undervaccinated. The latter refers to HCWs not receiving all three of the required doses,^9^ or receiving all three doses but, because of potential poor immune response, requiring an antibody titre to see if they mounted enough antibodies for protection.^10,11^ Poor immune response to vaccination can be attributed to old age, diabetes mellitus, other viral infections, and low body mass index, all of which contribute to weak immunogenicity. Because of these multiple factors, the World Health Organization (WHO) recommends compulsory vaccination for HCWs that includes additional monitoring of their immune response after vaccination.^9–13^

In Tanzania, the prevalence of HBV infection among HCWs in tertiary hospital in Mwanza was found to be 7.0%.^14^ This prevalence is similar to a study of HBV infection among blood donors in Dar es Salaam, Tanzania, which found 8.8% prevalence in that study population.^14^ Low knowledge of the risks for HBV infection place medical laboratory workers at risk for and low vaccination coverage among HCWs increases the risk of acquiring HBV infection following occupational exposure. The aim of our study is to provide information on the seroprevalence and vaccination coverage of and risk factors for HBV infection among HCWs at the Kilimanjaro Christian Medical Centre (KCMC) referral hospital in Moshi, Tanzania.

## METHODS

### Study Site and Design

KCMC is a tertiary referral and teaching hospital in the Kilimanjaro region of Tanzania. It is 1 of the 4 zonal consultant hospitals in the country, and is the referral hospital for over 15 million people in Northern Tanzania.^15^ The centre's laboratory is vast complex with multiple units that specialise in different clinical areas and employs 85 people. A cross-sectional study was conducted from January to June 2014, involving HCWs who work in the centre. Study participants were recruited from clinical and biotechnology laboratories, which provide services to all patients coming to the centre.

### Methods and Tools

The study conducted questionnaires with and collected biological samples from HCWs who met the study's inclusion criteria. The principal investigator completed the structured questionnaire for each HCW included in the study. The questionnaire had 4 sections: demographic and academic characteristics, knowledge of the risk factors for HBV infection and vaccines, history of accidental exposure to blood and its products, and the perception of HBV vaccines and vacci-nation status. A detailed history was obtained from each HCW about their HBV vaccination history and any occupation risks, such as exposure to infected blood through a splash, needle stick, or cut/wound. Other invasive procedures, such as intravenous therapy, intramuscular or subcutaneous injections, blood transfusions, and surgery were recorded. Identity (ID) numbers were written or placed on each participant's questionnaire and blood container to ensure the questionnaire and blood sample were matched to the same person.

### Study Participants and Sampling

To be eligible to participate in the study, HCWs must have been employed for more than 3 months, able and willing to provide consent, and willing to complete the self-administered questionnaire. Recruitment was non-randomised; a simple and consecutive sampling of eligible HCWs was conducted at the hospital during study period. The KCMC laboratory had total number of 85 workers, of which 3 did not consent, 3 had been employed for less than 3 months, and 3 did not complete the questionnaire, and were therefore excluded from the final study population of 76 HCWs. Every consenting participant was given a hard copy of the questionnaire to complete at their will and convenience. Participants were given a unique ID number which was used in place of their names, allowing the questionnaires and blood samples to be paired and submitted anonymously to the researchers.

### Blood Collection

Blood (4 mL) samples were obtained by venepuncture from all consenting participants using sterilised disposable 5 mL syringes and 20 gauge needles. A red top bottle was labelled with the study participant's specific study ID number. An hour after the blood samples were drawn, they were processed at the KCMC clinical laboratory.

### Sample Processing

All collected blood samples were tested for the hepatitis B antigen (HBsAg) using enzyme linked immunosorbent assay (ELISA) according to the manufacturer's guidelines. Serums/plasma were added to antigens or antibodies fixed to a solid surface microplate, incubated, and then washed using Thermo Scientific Wellwash Versa. The samples were then taken to Thermo Scientific Multiskan FC microplate photometer reader, with Skanlt Software, to be scanned and processed. To ensure this process provided correct results, rapid tests for HBsAg were used to re-test any positive test for HBsAg. All the methods used were in accordance with government laboratory standards. In the context of the current study, a person was considered to be fully vaccinated after receiving a minimum of intramuscular injection of 1 mL of DNA-recombinant vaccine scheduled at 0, 1, and 6 months, thus completing the minimum of the required 3-vaccine series.^16^ Participants who did not receive the 3 doses were considered to be inadequately vaccinated or undervaccinated, while those who did not receive the single dose were considered unvaccinated.

### Statistical Analysis

Data were captured in Microsoft Excel and transferred to Stata version 13.1 (StataCorp, College Station, Texas, USA) for analysis. Descriptive analysis was performed whereby numeric variables were summarised using measures of central tendency and the corresponding measures of dispersion. Categorical variables were summarised using frequency and percentages. Chi-square (X^2^) test was used to compare the prevalence of hepatitis B infection by different participant characteristics.

Multivariable logistic regression (odds ratio and the corresponding 95% confidence interval) was used to determine factors associated with hepatitis B infection among HCWs at KCMC. A cut-off point of 10% (*P*>0.1) was used to select variables to be included in the further analysis. After controlling for potential confounders, variables with *P*<0.05 were considered to be statistically associated with hepatitis B infection.

### Ethical Approval

The study was conducted following ethical approval from the Kilimanjaro Christian Medical University College Review Board and approval from the medical director of KCMC. Written informed consent was obtained from all participants.

## RESULTS

Among the 76 study participants, 40 (52.6%) were men and 36 (47.4%) were women ages 20–56 years with a duration of employment ranging from 3 months to 29 years. Health histories of the group revealed that 8 (10.5%) had a history of vaccination, 7 (9.2%) had a history of blood transfusion, 5 (6.6%) had history of mucocutaneous exposure to HBV, and 3 (3.9%) had history of surgery. Among the 9 (11.8%) HCWs who tested positive for HBsAg from unvaccinated group, 6 (66.7%) were medical personnel, 2 (20.0%) were administrators who had previous history of surgery and blood transfusion, and 1 was an information technology (IT) specialist. The characteristics and distribution of participants with HBV positive and negative results are summarised in Table 1. The frequency of hepatitis B infection among medical personnel was 9.1%, while among non-medical personnel was 30%. In this study, only 7 HCWs had been vaccinated against HBV – 4 received full vaccination coverage, 2 received only 2 shots, and the last received only 1 shot of the vaccine. None of the workers had their antibody status checked after finishing the full 3 shots of vaccine, which is WHO protocol.

**TABLE 1.** Positivity and Risk Factors of Occupational Exposure in Health Care Workers at KCMC, Moshi Tanzania

			Reported ‘Yes’ for Below Potential Exposures of Infection							
Characteristics	Number	Positive Hepatitis B Surface Antigen n (%)	Blood Transfusion n (%)	IV/IM n (%)	Operation n (%)	Muco-cutaneous Exposure n (%)	Vaccinated n (%)	Knowledge on HBV InfectionandVaccination		
								Low n (%)	Moderate n (%)	High n (%)
Sex										
Male	40	4 (10.0)	3 (7.5)	5 (12.5)	1 (2.5)	4 (10.0)	5 (12.5)	6 (15.0)	15 (37.5)	19 (47.5)
Female	36	5 (13.9)	4 (11.1)	8 (22.2)	0 (0.0)	3 (8.3)	2 (5.6)	7 (19.4)	24 (66.7)	5 (13.9)
Age (Years)										
<40	49	4 (8.2)	4 (8.2)	1 (2.0)	3 (6.1)	6 (12.2)	3 (6.1)	9 (18.4)	27 (55.1)	13 (26.5)
≥40	27	5 (18.5)	3 (11.1)	12 (44.4)	0 (0.0)	1 (3.7)	4 (14.8)	4 (14.8)	12 (44.4)	11 (40.7)
Cadre										
Medical personnel	66	6 (9.1)	5 (7.6)	13 (19.7)	1 (1.5)	7 (10.6)	7 (10.6)	8 (12.1)	34 (51.5)	24 (36.4)
Non-medical personnel	10	3 (30.0)	2 (20.0)	0 (0.0)	2 (20.0)	0 (0.0)	0 (0.0)	5 (50.0)	5 (50.0)	0 (0.0)
Duration at Work (Years)										
≤5	44	4 (9.1)	4 (9.1)	2 (4.5)	2 (4.5)	2 (4.5)	2 (4.5)	9 (20.4)	25 (56.8)	10 (22.7)
>5	32	5 (15.6)	3 (9.4)	11 (34.4)	1 (3.1)	2 (6.3)	5 (15.6)	4 (12.5)	14 (43.7)	14 (43.7)

Abbreviations: KCMC, Kilimanjaro Christian Medical Centre; IV, intravenous puncture; IM, Intramuscular puncture. Notes: Knowledge was measured by asking 20 standardized questions. Each question carries 5 marks: Therefore 5X20=100%. Grading knowledge basing on the obtained scores by the study participants (1) Low knowledge ≤40% (2) Moderate knowledge 41-65% (3) High >65%

The questionnaire given to participants assessed their knowledge of HBV infection and vaccination, scores were grouped into low (<50%), moderate (50%–74%), and high (≥75%) categories (Table 1).^17^ Knowledge about HBV infection and vaccination was higher among men compared to women. Knowledge was higher among medical personnel and very low among non-medical personnel – about 5 (50%) of all non-medical personnel had low knowledge about HBV infection and vaccination, as compared to 8 (12%) of medical personnel (Table 1).

On assessing the risks, out of the 66 medical personnel studied, 6 (9.1%) were positive for HBsAg; and among those 6, 5 (83%) had positive history of blood transfusion and only 1 had had surgery. For non-medical personnel, 3 were positive for HBsAg and 2 (67%) had a positive history of blood transfusion.

## DISCUSSION

The current study revealed a significant burden of HBV infection among HCWs at the KCMC referral and teaching hospital in Moshi, Tanzania. The overall seroprevalence of HBsAg among HCWs at KCMC was 11.9%. This prevalence is higher than a similar study conducted in Tanzania's Lake Zone, which found 7% prevalence among HCWs – doctors, medical laboratory workers, nurses, IT experts, among others.^14^ In the neighbouring country of Uganda, a similar study was conducted among HCWs, which revealed a prevalence of 8.1%.^4,14^ Among the 6 medical attendants who participated in the KCMC study, 3 (50%) were infected with HBV, most of whom scored very low on hepatitis B knowledge on the questionnaire. This may be explained by their higher level of exposure to infected material, such as used needles and soaked gauze, which increased their risk of infection; and by their low knowledge of the disease and its risk factors, which may have affected their behaviour towards using protective gear to reduce their risk.

In this study, potential risk exposures among HCWs, as a whole, might be among the predisposition to occupational accidents, as reported in other studies.^16–19^ However, of the 5 IT specialists in our study, 2 were infected with HBV. This could be explained by the fact that they receive and enter blood sample information into the laboratory information system, which exposes them to the blood products. Other possible explanations for their level of infection is their exposure to HBV through blood transfusion and surgery or their poor knowledge about HBV infection and vaccination. Although the study had limited information about their immune status information prior to employment, occupational risk factors for contracting HBV in the laboratory environment cannot be completely excluded. The incidence of occupational exposure is different from one facility to another depending on the quality of facility and knowledge of the workers as well as the prevalence of HBV in the general population.^18^

The current study found that a quarter of the study participants were above 40 years of age. Most of the HCWs who were infected were from this group. One explanation for this may be that HCWs have greater exposure to HBV during their lifetime; hence, prevalence may increase with age. Another explanation, which is consistent with other studies, is that long occupational exposure – number of years of employment – increase chances for HBV acquisition.^14,20^

To date, Tanzania has not performed a controlled and monitored nationwide vaccination campaign among high-risk groups, such as HCWs, as recommended by WHO.^16^ Very few HCWs have been vaccinated because vaccinations were only given as a donation or as a precondition for clinical research work.^4,21^ This study assessed vaccination coverage among HCWs and found it to be very low, which is also seen in other developing countries. The KCMC study's level of vaccination coverage among HCWs was 8.8%. In contrast, vaccination coverage of medical students in Cameroon was 18%; however, only 10% of the fully vaccinated students had their antibody levels checked.^20,21^ In this study, of the few who had been vaccinated, some did not finish the full vaccine series; and none of the HCWs who did finish the full dosage series, had an antibody titre to confirm immunity, which is recommended by WHO. A study in Karachi reported a high vaccination coverage of 81.8%,^22^ however, the study population's level of immunity was not confirmed with an HBsAg titre.

The KCMC study assessed the level of knowledge on HBV infection and vaccination among HCWs, which was found to be generally very low. Good knowledge about HBV vaccination and infection may reduce infection rate. A study conducted in the Sudan showed that some medical laboratory workers did not know that HBV can lead to liver cancer, they only knew about the relationship between cirrhosis and liver cancer.^23–25^ This suggests more training is needed for HCWs on HBV infection and vaccination.

In the KCMC study, knowledge was higher among medical personnel compared to non-medical personnel. This could be attributed to difference in the type of education each group received. However, some cases of low knowledge among medical personnel have been reported; for example, literature from Iran showed poor knowledge among medical doctors about HBV infection occurring through vaginal route.^26,27^

We assessed different factors, such as surgery and blood transfusion, that contributed to HBV infection. Out of the 6 medical and 3 non-medical personnel who tested positive for HBV, 5 and 2, respectively, had history of blood transfusion. These factors likely contributed to the high prevalence among these particular participants. A study conducted in Gabon showed the contribution of blood transfusion and surgical interventions towards increasing seroprevalence of HBV.^28^ Our study found no difference in positivity between participants with many years of work experience compared with those workers with only a few years.^29^

## Limitations

Due to small sample size of our study, which focused only to personnel within a laboratory setting, our findings are not generalizable to HCWs in other settings. All information about HBV vaccination status was obtained through interviews without laboratory confirmation of an antibody titre, which may have introduced the possibility of interviewee bias.

## CONCLUSIONS

This study showed high prevalence of HBV and low coverage of HBV vaccination among laboratory workers. Low knowledge of risks for HBV infection may have placed these workers at great risk of occupational exposure. Low vaccination coverage among HCWs increases the risk of acquiring HBV infection following occupational exposure. The aim of this study was to provide a further evidence of a major problem that needs immediately attention and action to be taken. Tanzanian government has done great work for HBV vaccination among children under 5; we suggest the same calibre of effort must be considered for workers in all specialties.
